# Supplementary material for: ARID1A loss is associated with increased NRF2 signaling in human head and neck squamous cell carcinomas
Source: PLoS One. 2024 Feb 15;19(2):e0297741. doi: 10.1371/journal.pone.0297741 (PMC10868765; doi:10.1371/journal.pone.0297741)
Supplement: Attachment — Submitted filename: Response to Reviewers_2024_01_05.docx [file pone.0297741.s007.docx]

Journal requirements:

1. Please ensure that your manuscript meets PLOS ONE's style requirements, including those for file naming.

*The manuscript was edited by the manuscript preparation expert in our Cancer Center. She found multiple errors and corrected them. We apologize for not catching them before the initial submission.*

2. Please note that PLOS ONE has specific guidelines on code sharing for submissions in which author-generated code underpins the findings in the manuscript. In these cases, all author-generated code must be made available without restrictions upon publication of the work.

*We acknowledge and agree with this policy.*

3. We note that the grant information you provided in the ‘Funding Information’ and ‘Financial Disclosure’ sections do not match.

When you resubmit, please ensure that you provide the correct grant numbers for the awards you received for your study in the ‘Funding Information’ section.

*We have corrected this issue.*

4. Thank you for stating the following financial disclosure:

"This work was supported by the National Cancer Institute [R01 CA216051] (MBM & BEW), the National Institute of Dental and Craniofacial Research [K08 DE029241] (TS) and the National Institute of Environmental Health Sciences [T32 ES007126] (VN)"

Please state what role the funders took in the study. If the funders had no role, please state: ""The funders had no role in study design, data collection and analysis, decision to publish, or preparation of the manuscript.""

If this statement is not correct you must amend it as needed.

Please include this amended Role of Funder statement in your cover letter; we will change the online submission form on your behalf.

*The statement “The funders had no role in study design, data collection and analysis, decision to publish, or preparation of the manuscript.” is correct. We have added this statement to the Cover Letter.*

Reviewers’ comments:

Reviewer #1: Title: ARID1A loss is associated with increased NRF2 signaling in human head and neck
squamous cell carcinomas.

This is a very nicely performed study. The authors demonstrated how NRF2 and SWI/SNF mutations are related to human malignancies, and they suggested that ARID1A inactivation in HPV-negative HNSC promotes tumour growth and survival by activating the KEAP1-NRF2 signalling pathway. Overall, this is a quite solid paper that provides clear evidence.

I have a few minor comments and hope they help the authors.

1. The full stop is missing in many sentences. Please correct this throughout the manuscript.

*We again apologize for the grammatical errors. We have corrected them in the revised manuscript.*

2. The Limma-voom pipeline was used for all subsequent differential expression analysis(31)'. Why did the authors choose the Limma loop pipeline in place of DeSeq as DeSeq finds more differentially expressed genes?

*Deseq2 differential expression function accepts raw count matrices. However, we desired to perform differential expression followed by defining classifiers based on the nearest centroid method. For centroids to be reasonable, statistical constructs library size normalization is needed. Limma-Voom allowed us to work with the same set of pre-processed (Normalized LCPM) expression values for both the differential expression, as well as the related centroid classifier. This approach seemed ideal as the differential expression step principally suggested genes/features that would perform well as the basis for a classifier. With this initial motivation, all other differential expression-based analyses (e.g., volcano plots, heatmaps) in the paper were performed with Limma-Voom for methodological unity.*

Reviewer #2: In this study, author explored the relationship between mutations in BAF and PBAF subunits of the SWI/SNF complex and activation of NRF2 signal transduction by open source data. However, these conclusions only supported by data of DNA and RNA level. We did not find out the mechanism or clinical significance from this paper.

*This study was designed as a bioinformatic query of the potential relationship between loss of ARID1A expression and activation of NRF2 signaling. The results from this manuscript lay the groundwork for the more extensive in vitro and in vivo biological and molecular studies required to identify underlying mechanisms.*

*We also added a new paragraph at the end of the Discussion to emphasize the clinical significance of NRF2 activation and ARID1A in patients with HNSC.*
